# Cutaneous Sarcoidosis: A Differential Diagnosis to Consider in Undiagnosed Skin Lesions

**DOI:** 10.7759/cureus.39852

**Published:** 2023-06-01

**Authors:** Sumona Islam, MD Sabbir Hossain, Khaled M Murshed, Mohammad Ferdous Ur Rahaman, MD Abul Kalam Azad

**Affiliations:** 1 Department of Gastroenterology, Bangabandhu Sheikh Mujib Medical University, Dhaka, BGD; 2 Department of Internal Medicine, Bangabandhu Sheikh Mujib Medical University, Dhaka, BGD

**Keywords:** refractory skin lesions, lung fibrosis, skin biopsy, noncaseating granuloma, cutaneous sarcoidosis

## Abstract

The presentation of sarcoidosis varies depending on the organs involved. Cutaneous sarcoidosis usually presents with other organ involvement, but isolated presentation is possible. However, diagnosing isolated cutaneous sarcoidosis can be challenging in resource-poor countries, particularly where sarcoidosis is relatively uncommon, since cutaneous sarcoidosis usually does not cause troublesome symptoms. We present a case of cutaneous sarcoidosis in an elderly female who had been suffering from skin lesions for nine years. The diagnosis was made after the appearance of lung involvement, which raised the suspicion of sarcoidosis and prompted a skin biopsy. The patient was then treated with systemic steroids and methotrexate, and her lesions improved shortly thereafter. This case highlights the importance of considering sarcoidosis as a possible cause of undiagnosed, refractory cutaneous lesions.

## Introduction

Sarcoidosis is an idiopathic disease characterized by granulomatous inflammation affecting multiple organs in the body [1]. The lungs are the most frequently involved site, accounting for approximately 90% of cases [2]. Cutaneous involvement is observed in 25%-30% of patients; however, less than one-third of those with cutaneous manifestations present with isolated skin involvement, without any systemic features [3,4]. Cutaneous sarcoidosis, like sarcoidosis in general, exhibits a higher prevalence among females [3,5]. Additionally, sarcoidosis is less frequently observed among individuals of Indo-Pakistani descent, which often contributes to delayed diagnosis, particularly when patients present solely with skin manifestations and no systemic involvement [6]. In this case report, we present an elderly female who suffered from cutaneous lesions affecting multiple regions of the body for a prolonged period of nine years, without a definitive diagnosis. Subsequently, she was diagnosed as a case of sarcoidosis.

## Case presentation

Nine years ago, our patient, a 59-year-old hypertensive, nondiabetic female, noticed plaque-like lesions on her forehead that gradually spread to her upper back and chest. The lesions were itchy but not painful or photosensitive. The patient sought advice from several physicians and received both over-the-counter and prescribed medications. While the lesions varied in size over the years, they never disappeared. Despite being advised to undergo a skin biopsy twice, the patient chose not to do so because the lesions were not painful and responded partially to over-the-counter medications, including topical steroids.

This time, the patient presented to us with her usual skin lesions, along with a new complaint of progressively increasing dyspnea for the last 18 months. Initially, there was a mild cough and exertional dyspnea, but it has now progressed to interfere with daily activities. The cough does not follow any specific diurnal or seasonal pattern. The patient reports no fever, night sweats, joint pain, oral ulcers, chest pain, or difficulty in swallowing. There is no history of past tuberculosis (TB), smoking, biomass fuel use, or living with pets. Furthermore, there are no other significant personal or family medical histories.

**Figure 1:**
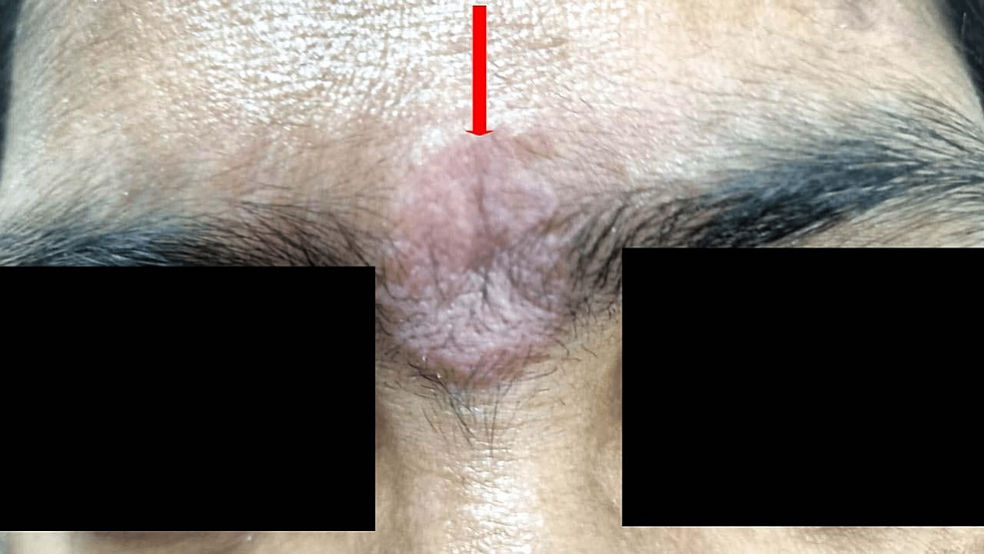
Erythematous plaque in the forehead

**Figure 2:**
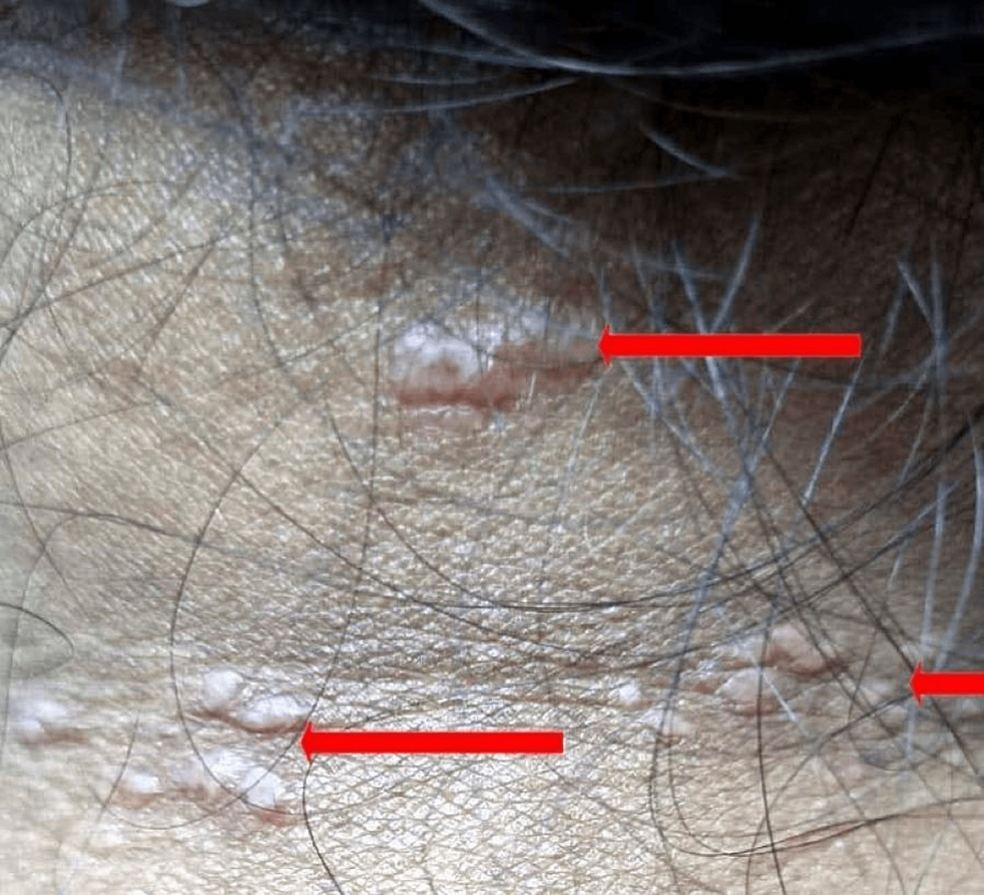
Multiple papules and plaques on the nape of the neck

During the skin examination, numerous well-demarcated erythematous papules and plaques of different sizes were observed on the forehead, upper back, and chest, ranging from 2 to 8 cm in diameter (Figures 1-2).

Sensation over the affected areas was normal, and the Auspitz sign was negative. The examination of other areas, including the lymph nodes, oral cavity, and genitalia, did not reveal any significant abnormalities. Respiratory system examination revealed fine bilateral basal end-inspiratory crepitations, which were unaltered by cough. Other systemic examination revealed no abnormalities.

**Figure 3:**
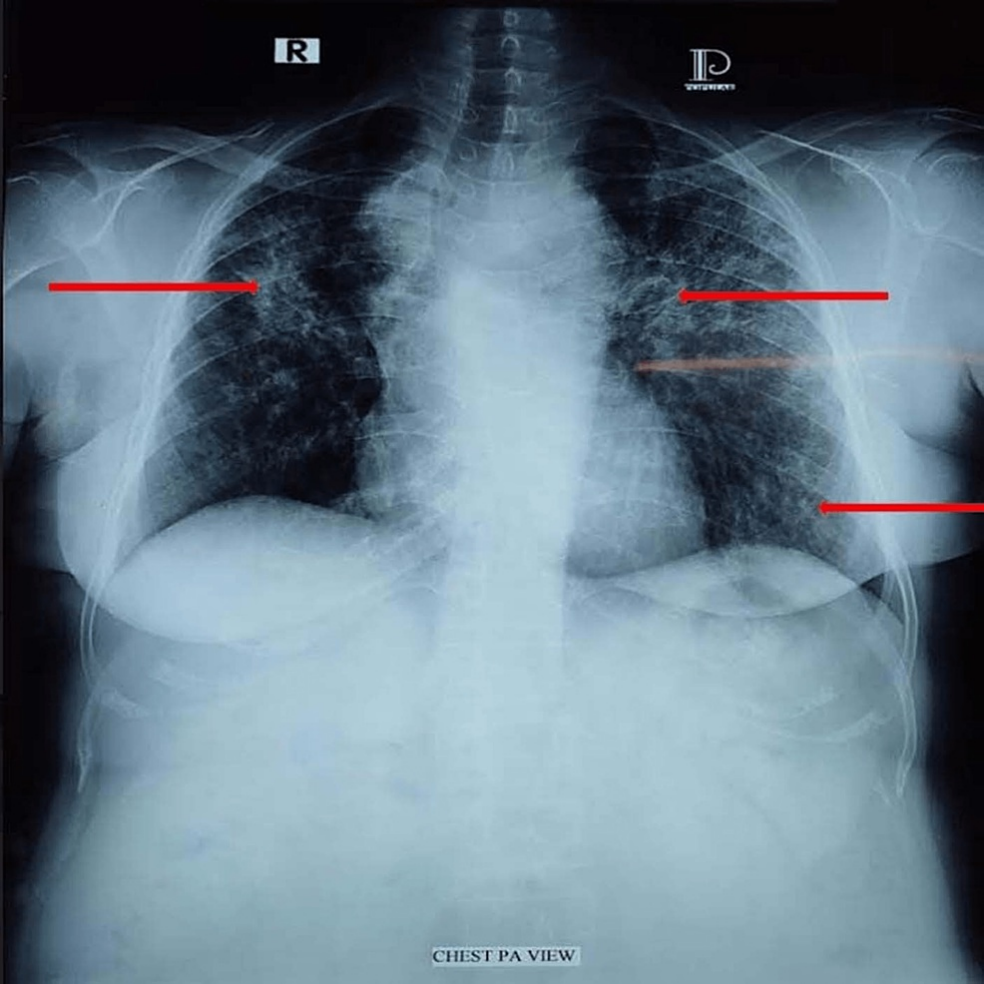
Anteroposterior chest X-ray image showing homogeneous opacities over the upper, middle, and lower zones of both lung fields

Considering the patient's long-standing skin lesions and the presence of crepitations over both lungs, sarcoidosis became a suspected diagnosis. To further investigate, chest X-ray was done, which showed homogeneous opacities over the upper, middle, and lower zones of both lung fields (Figure 3).

**Figure 4:**
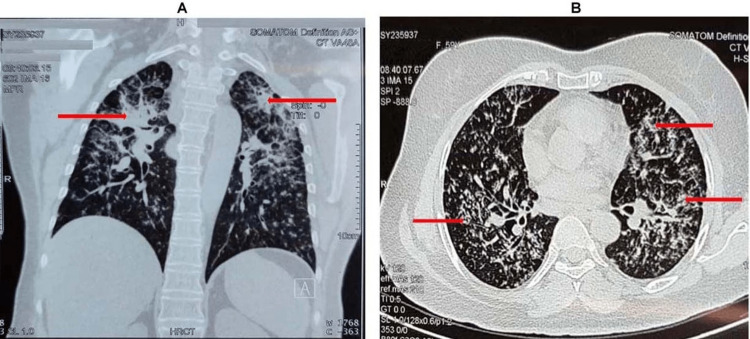
High-resolution CT scan of the chest showing diffuse reticular opacity affecting all segments of both lungs A, coronal view; B, axial view

The chest CT scan revealed diffuse reticular opacity with thickening of interlobular septa and fibrosis affecting all segments of both lungs (Figure 4A-4B).

Pulmonary function tests indicated a restrictive pattern of lung disease with a mild reduction in diffusing capacity for carbon monoxide (DLCO). The six-minute walk test was normal. Bronchoalveolar lavage showed no evidence of bacterial or fungal infection. The corrected calcium level was measured at 10.26 mg/dl, indicating mild hypercalcemia. Additionally, the serum angiotensin-converting enzyme (ACE) level was elevated at 138 U/L. These findings support the possibility of sarcoidosis as a potential diagnosis.

**Figure 5:**
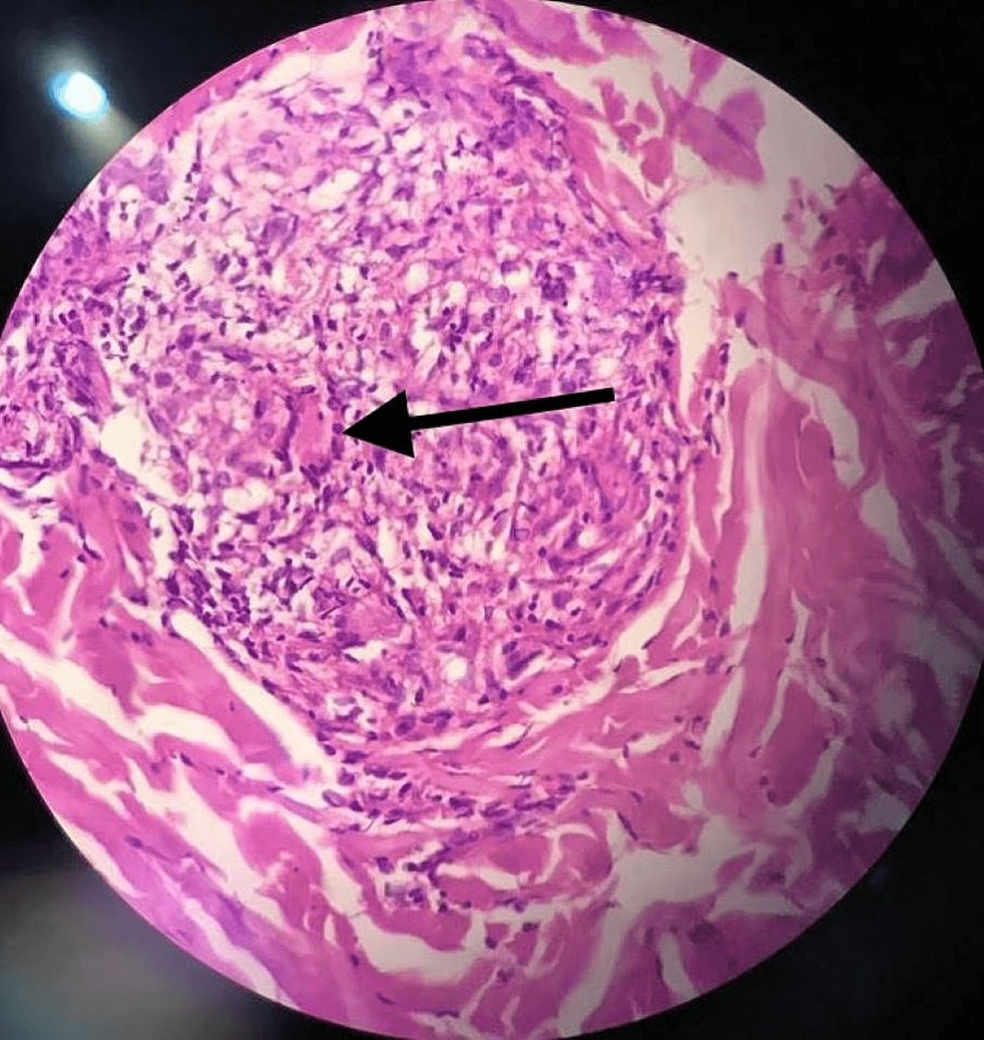
High-power view of the skin biopsy High-power view demonstrates multinucleated giant cell (arrow) and epitheloid histiocyte

A punch biopsy of the skin lesion, stained with hematoxylin and eosin, demonstrated the presence of non-caseating granulomas composed of epithelial cells, foamy histiocytes, and a small number of lymphocytes in the dermis and subcutaneous tissue (Figure 5).

Fite-Faraco stain, used to detect acid-fast bacilli (AFB), yielded negative results.

Based on the aforementioned findings of elevated serum angiotensin-converting enzyme (ACE) and mild hypercalcemia, along with the presence of non-caseating granuloma on skin and lung fibrosis, we reached a diagnosis of sarcoidosis with the involvement of both the skin and lungs.

The patient was prescribed oral prednisolone at a daily dose of 30 mg, along with methotrexate at a weekly dose of 10 mg. Following two months of treatment, the skin lesions completely resolved, and the severity of cough and dyspnea decreased. At this point, the steroid was gradually tapered.

## Discussion

Sarcoidosis is a multisystem disorder of unknown etiology, likely influenced by genetic factors and exposure to environmental triggers [5]. While lung involvement is common, affecting around 90% of cases, cutaneous involvement is also prevalent, albeit often accompanied by other organ manifestations [2-4]. The most frequently observed cutaneous lesions in sarcoidosis are plaques and papules. Additionally, other types of lesions such as macules, nodules, and lupus pernio can also occur [5,7,8]. The trunk, face, and upper extremities are the most frequently affected sites [3].

Sarcoidosis is less common in South Asian countries, which translates to it rarely being considered a differential diagnosis for cutaneous lesions in this region [6]. Similarly, our patient experienced nine years of skin lesions without sarcoidosis being considered until lung involvement developed. Additionally, since cutaneous sarcoidosis is rarely troublesome, patients may be hesitant to undergo an invasive procedure such as a skin biopsy.

Another notable challenge arises from the fact that sarcoidosis, which causes granulomas, shares similarities with tuberculosis (TB). A case report highlights the diagnostic complexity, where an Indian man presented with granulomas on lung biopsy and was initially treated with anti-TB medications, only to be later diagnosed with sarcoidosis [9]. This problem is a real challenge in TB-endemic zones.

Sarcoidosis with systemic involvement is often linked to increased serum ACE levels, while isolated cutaneous involvement typically presents with normal ACE levels [7]. Our patient, however, exhibited elevated serum ACE levels and lung involvement. However, it remains uncertain if the ACE level was elevated before the lung involvement developed, which holds significant relevance. When suspecting cutaneous sarcoidosis, serum ACE levels do not provide any clue. A clinical suspicion should be raised, and a biopsy of the skin lesion is advised for a proper diagnosis.

The standard treatment for cutaneous sarcoidosis typically involves topical corticosteroids or intralesional steroids. However, in cases where these treatments are ineffective, systemic steroids may be used in conjunction with methotrexate or antimalarial drug [10]. In our case, we administered oral steroids along with methotrexate to manage the condition.

## Conclusions

Our patient presented with plaque-like lesions, which are the most common type of lesion in sarcoidosis, particularly in the face, neck, and upper extremities, which are the usual sites for sarcoidosis. However, despite experiencing these lesions for a prolonged period of nine years, the diagnosis was not made. This case highlights the significance of considering sarcoidosis as a potential diagnosis for undiagnosed cutaneous lesions, particularly in resource-limited medical settings and regions where sarcoidosis is less prevalent.
